# Tensions in the therapeutic relationship: emotional labour in the response to child abuse and neglect in primary healthcare

**DOI:** 10.1186/s12875-022-01661-7

**Published:** 2022-03-17

**Authors:** Jacqueline Kuruppu, Cathy Humphreys, Gemma McKibbin, Kelsey Hegarty

**Affiliations:** 1 Department of General Practice, The University of Melbourne, Melbourne, Australia; 2 Department of Social work, The University of Melbourne, Melbourne, Australia; 3 The Royal Women’s Hospital, Parkville, Victoria Australia

**Keywords:** Child abuse and neglect, Child maltreatment, Primary care, Response, Mandatory reporting, Emotional labour, Therapeutic relationship

## Abstract

**Background:**

Child abuse and neglect (child abuse) is a prevalent public health issue linked to survivors experiencing a higher risk of health issues such as obesity, heart disease and major depression. Given the significant impact of child abuse on health, general practitioners (GPs) and primary care nurses (nurses) are well-placed to respond to child abuse. However, research shows that responding to child abuse is difficult for health practitioners, especially the act of reporting child abuse. The present study aimed to understand how GPs and nurses experience the response to child abuse in primary healthcare.

**Methods:**

This study employed qualitative methods. Twenty-six in-depth individual and group interviews were conducted with 30 GPs and nurses. The interviews were audio recorded with consent, transcribed verbatim and thematically analysed.

**Results:**

The participants were mostly metropolitan-based female GPs. Participants were sampled from two settings: private general practice and community health; and Doctors in Secondary Schools, a program that places GPs and nurses in high schools. Thematic analysis generated four themes: *blowing trust out of the water; riding the reaction wave; opening a hornet’s nest;* and *battling emotions.* Participants felt that, in considering child abuse, they were betraying the trust of the therapeutic relationship and thus, had to manage their patients’ reactions to preserve the therapeutic relationship. They used strategies that created shifts in perception in both themselves and their patients to help maintain the therapeutic relationship. Participants often felt that they had to compromise their professional code of ethics to fulfil their mandatory reporting obligations. Thus, they experienced internal emotional battles when responding which led to some experiencing burnout or vicarious trauma and others resilience. This complex interplay of relationship and emotional management was placed in the context of emotional labour theory. We contend that our participants undertook emotional labour across three levels: internal, organisational and systemic.

**Conclusions:**

We conclude that the emotional labour exerted in the response to child abuse can be diminished by: developing strategies for therapeutic relationship management; undertaking an internal, organisational and systemic values assessment; and facilitating communication between health professionals and the child protection system.

## Background

Child abuse and neglect (hereafter referred to as child abuse) is a significant public health issue affecting 50% children and young people aged between two and 17 years globally [1]. It is defined as any intentional or unintentional act or omission of care by a person in a position of power over a child that results in harm, the potential for harm or the threat of harm to a child [2, 3]. There are five recognised types of abuse: physical, sexual and emotional abuse; neglect; and exposure to domestic violence [3]. The health impacts of child abuse can be acute, with children experiencing developmental delays, learning difficulties, behavioural problems and lower quality of life [4, 5]. These impacts are evident across the lifespan, with adult survivors more likely to experience physical health complications, serious depression, and suicidality [6–8].

Rivara et al. conducted a longitudinal cohort study in the US with 1391 children and found that children who had been exposed to domestic violence engaged with primary care more often than those who had not been exposed to domestic violence [9]. In Australia, general practitioners (GPs) and primary care nurses (nurses) see 83% of the population yearly [10]. Given this and the health impacts of child abuse, GPs and nurses occupy a prime position in the community to identify and provide early intervention for families experiencing child abuse.

Recognising the role of primary care, Australian state and territory governments rolled out legislation to mandate reporting of child abuse legislation over a 40-year period to protect the rights of children [11]. Mandatory reporting laws differ between Australian jurisdictions according to how ‘child’ is defined, what constitutes reportable abuse and who is mandated to report, although GPs and nurses are mandated to report in every Australian state and territory [12].

Medical personnel are the third most common source of notifications for investigated cases, following school personnel and police [2]. However, the process of reporting is emotionally challenging for health professionals [13–15]. Some health professionals have not reported child abuse in order to avoid the emotional burden associated with reporting [16]. A qualitative study with 6 US primary care physicians found that experiences of identifying and reporting child abuse were emotionally-charged and characterised by a burden of responsibility [17]. Further, similar qualitative findings have placed the emotional burden of reporting child abuse in the context of emotional labour theory [18].

Emotional labour comprises internal emotional labour and organisational emotional labour in the context of work [19, 20]. Internal emotional labour refers to the way in which employees manage their internal emotion states, including engaging in ‘surface acting’ where employees modify facial expressions and manner in order to serve the goals of the organisation [19–21]. For example, a GP experiencing exhaustion might feign an energetic persona when interacting with a patient. Employees may also engage in ‘deep acting’ where they change patterns of thinking over time to manage their emotion states to suit the goals of the organisation [19, 20, 22]. Organisational emotional labour concerns work demands regarding emotional expression [19, 20]. For example, a GP or nurse is expected to exhibit caring emotions in their line of work and maintain a therapeutic relationship. When the internal feeling state of an employee is not aligned with the emotional demands of an organisation, an employee can experience emotive dissonance whereby the worker is forced to portray feelings they do not feel to meet organisational goals [19, 21]. The continual surface acting associated with emotive dissonance has been linked to burnout, with deep acting not being associated with emotional exhaustion [20, 23].

Work-related burnout is indicated by the presence of exhaustion, cynicism and inefficacy that emerges over time in response to chronic emotional and interpersonal work stressors [24]. It is characterised by poor boundaries, guilt, low energy or depression [24]. Research has explored the link between burnout and vicarious trauma, the latter of which differs from burnout by being characterised by experiencing PTSD-like symptoms in response to traumatic material. Trippany, Kress and Wilcoxon proposed that the relationship between burnout and vicarious trauma develops over time [25], however, this relationship is not strongly established in the literature [24].

While the emotionally challenging nature of reporting is well acknowledged in the literature [13–15], the contributors to this emotional challenge have mostly been explored using a cross-sectional survey design [15] and thus fail to capture the richness, complexities, and nuance of such a highly emotional topic. The emotional challenges within the context of the broader response to child abuse, including but also beyond reporting, have not yet been explored. Further, the emotional labour involved in maintaining the primary care therapeutic relationship in the context of responding to abuse has yet to be explored. Since so little qualitative research has been conducted on this topic, we decided that our study would be exploratory in nature. Therefore, qualitative methods were used for this study which aimed to deeply understand how GPs and nurses experience the response to child abuse. We sought to achieve this aim by answering the following research question: What are GPs’ and nurses’ experience of the response to child abuse and neglect in primary healthcare settings?

## Methods

### Study design

Individual and small group interviews were conducted as the intimacy of this setting allowed for greater flexibility in exploring the response to child abuse. This study design was underpinned by a social constructivist paradigm or belief that there is no single truth, rather there is subjective truth created by experience and social interaction [26], and a hermeneutic phenomenology methodological perspective, which explores lived experience and interprets the meaning it brings [26–28].

### Participants and recruitment

GPs and nurses were recruited via convenience, purposive and snowball sampling from two different settings described below. GPs and nurses were eligible to participate if they: had ever suspected child abuse and neglect; worked or were receiving specialised training in general practice; or worked in the Doctors in Secondary Schools program described below. A sampling frame was constructed to direct purposive sampling to achieve greater demographic diversity in the areas of gender, practice setting and profession.

### Context setting 1: private general practice and community health centres

Participants from this setting were GPs or nurses who worked only in private general practice or community health centres and answered the interview questions exclusively from this perspective. These participants were accessed via existing networks within the Sexual Assault and Family Violence team and the Department of General Practice. Several methods were used to advertise the study across these existing networks including placing advertisements in e-bulletins, newsletters, the Primary Health Networks (PHN) website and a GP-specific Facebook group. Additionally, emails advertising the study were sent directly from the research team to known GPs interested in family violence research. GPs and nurses recruited from this setting were from anywhere in Australia.

### Context setting 2: doctors in secondary schools (DiSS)

The second setting from which participants were recruited was a program known as Doctors in Secondary Schools (DiSS) that places GPs and nurses in high schools for 1 day a week. Here, the GP and nurse work in partnership to run a clinic that students may visit without caregiver supervision during school hours for health issues. Students can self-refer or be referred by teachers or the school’s Wellbeing Team. Participants from this setting answered the interview questions drawing on their experience of working in DiSS and in private general practice. Some GPs and nurses had previous contact or initiated contact with some students’ families through their private general practice clinic. At the time of this study, DiSS only existed in Victoria, Australia. Advertisements were placed on DiSS portals and distributed via emails. Snowball sampling was particularly useful in this context as participants often recruited their colleagues working within their school clinic. In this paper, GPs and nurses working in Doctors in Secondary Schools are indicated with the acronym ‘DiSS’ appearing after their pseudonym. When this acronym does not appear, it indicates a GP or nurse who worked in private general practice or in a community health clinic.

Potential participants from either setting who were interested in participating were invited to email the first author to express their interest. Upon expressing interest, potential participants were emailed a plain language statement and a consent form. Once consent was obtained, the first author liaised with the participant to organise a time and place of the participant’s choosing to conduct the interview. Participants involved in group interviews chose with whom they completed the group interview (i.e. colleagues working within the same school setting). At the end of the interview, participants were asked to fill in a demographic information sheet.

### Data collection

The interview guide was informed by a scoping literature review [15]. The guide began with a preamble which contained some personal reflection by the interviewer to encourage the feeling of story exchange between interviewer and participant [29]. If the interviewer initially makes themselves vulnerable, participants may feel more comfortable to share sensitive stories [29]. The first question was a broad open-ended question about participants’ interest in child abuse. The questions then went on to explore experiences of suspecting child abuse, their response to this suspicion, the challenges of responding and what enabled or supported their response. The guide was piloted with colleagues at the Department of General Practice, and feedback was incorporated. The guide was also iteratively modified as data collection progressed, in line with our hermeneutic approach [30]. For example, a final question ‘what keeps you strong when dealing with such a difficult topic?’ was added to capture emotional enablers, finish the interview on a positive note and support the mental states of both the participant and the researcher.

Interview settings were informed by the participants’ choice, and were conducted: at the Department of General Practice, The University of Melbourne; at the participant’s general practice, community health or school clinic; or at informal locations such as participants’ homes or a park.

### Data analysis

All interviews were audio-recorded with consent and transcribed verbatim by the first author and a commercial transcription agency. Reflections on the interviews were recorded in field notes or in audio-recorded discussions with colleagues. All transcripts were de-identified during the transcription process and a pseudonym was chosen for each GP and nurse from a list of common names associated with each letter of the alphabet. Participants were given pseudonyms that reflected their given names but were sufficiently different from their given name to protect their identity.

The interview transcripts were imported into NVivo 12 (QSR International). The interviews were analysed thematically according to the reflexive thematic analysis framework outlined by Braun and Clarke [31]. After familiarisation with the data, the first author began initial line-by-line descriptive coding using an inductive approach for 10 of the transcripts. Each code was given a name using active language that closely resembled the line being coded. For example, this quote from a GP ‘*if I’m completely emotionally honest, I think for me, anyway – like I want to avoid the situation!’* was coded as ‘wanting to avoid the situation of child abuse and neglect’. The research team reviewed these codes and preliminary themes and a coding framework was developed. This coding framework was applied to all the remaining transcripts by the first author, with the second, third and fourth authors applying the framework to nine transcripts to ensure rigour. There were no disagreements in coding. Through the process of applying the framework and regular team discussions, the number and content of the themes shifted over time as they were iteratively developed. Table 1 contains a sample of quotes relating to each subtheme and theme.

**Table 1:** Theme Analysis

Theme	Subtheme	Example of text
**Blowing trust out of the water**	Betraying trust of the therapeutic relationship	*It’s a hard thing to do because you feel like you’re breeching the trust of the kid, on a lot of occasions.*
	Holding and balancing care	*But on the other hand, knowing the mother, she was desperate to have the children back and she’s apologetic and has put a lot of things in place to avoid the situation in future around her violence. So, I’m trying to support both the children and her.*
	Losing the therapeutic relationship	*But that was one of those times where that’s wrecked the therapeutic relationship with that family because they feel like police were called on them.*
**Riding the reaction wave**	Managing interaction with families	*You can’t just say well I think your kid is being abused and that’s the end of the consultation, I’m reporting this to the authorities. You’ve got to deal with the impact of that news… and not abandon them…it’s part of a fair degree of emotional energy to get engaged.*
	Personal fear of retaliation	*Well, I don’t feel anonymous. Um, I always tell people that I’m going to report, um, because I don’t, I don’t want someone turning up on their doorstep and then getting all shirty with me.*
	Fearing retaliation from organisation	*When someone is paying your fee, you’ve got to be a bit careful…if the parents are unhappy…they might take their business elsewhere and the word might get around.*
	Fearing retaliation from system	*God forbid I break a law, it’s scary thinking about breaking laws. I don’t want to get into trouble.*
**Opening a hornet’s nest**	Experiencing tension between professional ethics and mandatory reporting obligations	*And then you make that notification and then it goes nowhere. Or, it makes things worse. But nothing changes in the meantime…Or it escalates everything but there’s no further investigation from the department and so the kid’s not happy with us because we’ve made the report and frustrated because then nothing’s changed and then who do they trust?*
	Fearing for young people’s safety after making a report	*I’ll ring child protection…It kind of makes me feel really anxious, thinking ‘oh gosh, are they going to get the message? What are they doing? Is that young person okay?’'*
	Relationship between system, patients and GPs and nurses	*And then I think we feel pretty helpless sometimes. Some kids are quite severely injured or killed, and there was involvement of health professionals or DOCS. And you just feel like the system is crap and what’s the point? Not what’s the point, like why should I bother? …So you report something, you put someone in danger, and then the system can’t protect them.*
**Battling emotions**	Experiencing burnout and vicarious trauma	*I said to my GP it’s up to her… she’s burnt out because it is so tough because it’s all mental health. There is sexual abuse, there are transgender issues, there are alcoholic parents; it’s a big mess and she’s just taking a lot of it on.*
	Building resilience	*They’ve had to deal with quite so much, which is kind of inspiring in a way. I enjoy allowing them to gain inspiration from their own stories and from their own process to see what’s helped them to get through. So, I think that’s what helps to be able to continue doing that work.*

Ethics approval was obtained from the Human Ethics Advisory Group, Department of General Practice at the University of Melbourne (Ethics ID: 1851916.1).

## Results

Twenty-six individual and group interviews were undertaken with 22 GPs and 8 nurses. The interviews were, on average, 52 min long. The majority of the participants were female GPs who worked in metropolitan settings. There was a near even divide between the number of private and community health participants (*n* = 16) and DiSS participants (*n* = 14). Participant demographics are summarised in Table 2. Four themes were generated from the data: *blowing trust out of the water; riding the reaction wave; opening a hornet’s nest;* and *battling emotions*.

**Table 2:** Participant Demographics

	GPs (n)	Nurses (n)
Gender		
Male	5	0
Female	17	8
Age range^a^	38–66	35–50
Years in practice in Australian primary care^a^	0–38	8–30
Practice setting		
Metropolitan	18	5
Rural	4	3
Private general practice/community health	14	2
Doctors in Secondary Schools	8	6
State		
Victoria	15	8
New South Wales (NSW)	4	0
South Australia (SA)	2	0
Northern Territory (NT)	1	0
Queensland	0	0
Tasmania	0	0
Western Australia (WA)	0	0
Amount of family violence training^a^		
< 1 h	0	1
1–2 h	3	1
3–5 h	6	0
6–10 h	2	0
11–15 h	3	1
16–20 h	0	0
20h hrs	6	5

^a^Indicates missing data – 2 participants declined to share some demographic information

### Blowing trust out of the water

Betraying the trust of the therapeutic relationship was the aspect of response to child abuse that caused the greatest distress for most GP and nurse participants. This theme explores the feeling of betraying the trust of the therapeutic relationship, the conflicting obligations within the therapeutic relationship, and experiences of losing the therapeutic relationship.

For many GPs and nurses in this study, maintaining the therapeutic relationship and the trust it was built upon was paramount. Because of this, broaching the topic of child abuse and reporting was a confronting difficulty for GPs and nurses:

*‘GPs have a lot of problems raising that topic themselves. They know these people and …. all of a sudden you're going to throw this hand grenade into a long-term relationship you have with this patient and this family by basically blowing trust out of the water.’* Charlie, GP, NSW.

The feeling of betrayal was dual in nature. In considering child abuse, participants felt as if they had betrayed the trust of their patient. In addition, they felt it showed they had no trust in the patient themselves. Daniel, a GP who experienced a confrontation after asking a mother if it was possible her daughter was sexually abused, reflected on what the outcome would have been if the mother’s regular GP had expressed the same concern:

*‘But on the other hand…it might have been just as bad coming from him because the mum would have said ‘you know me, you trust me, you know what my family is like and then you still ask the question.”* Daniel, GP, SA.

Further complicating the matter of betraying trust was the act of holding and balancing care for the entire family. GPs and nurses in this study often experienced conflicting priorities throughout the response to child abuse. This meant that trust could be broken on multiple levels within the one family. For example, mandatory reporting law compelled some GPs and nurses to choose between supporting caregivers using abusive behaviours and the young people experiencing abuse. This forced choice often resulted in a termination of the relationship with some or all the family:

*‘I was trying to support the mother as my patient, while protecting the child…So, I had really a conflicted relationship…In the end I had to, obviously, prioritise the child…given my need to protect the child, there was no way I could support the mother in that situation.’* Willow, GP, Victoria.

Note that Willow identifies the mother who was using abusive behaviour as her primary patient, rather than the child. Some experiences within DiSS emphasised the difficulties involved in keeping the young person in view within the therapeutic relationship. Tara, who was initially treating a suicidal young person independent of their family within DiSS, explained how she ended up supporting each member of the family in her private practice who were experiencing health conditions ranging paranoid schizophrenia to uncontrolled diabetes:

*‘They* [the parents] *were incredibly selfish upon reflection. But, because I was trying to also treat the parents, I had this compassion for the parents as well, but then in the end I just kind of lost the plot with them* [the parents] *and I haven't seen them* [the parents] *for a while.’* Tara, GP, DiSS.

While DiSS is an uncommon practice setting for GPs and nurses, this experience exemplifies the way in which many GPs experience difficulties in keeping the young person in view. The health focus initially shifted from the young person to the parents and then finally pivoted back to the young person. When considering the possibility of abuse, GPs and nurses seem to make a choice, forced or otherwise, in favour of protecting and supporting the young person. Holding and balancing care for a family divided by abuse and violence meant that both the trust of the caregivers and the trust of the young person could be betrayed. This often resulted in competing loyalties which implied the question: Who’s really my patient?

Once trust was destabilised or destroyed, the therapeutic relationship often suffered a breakdown, or was lost altogether. The ramifications of loss of the therapeutic relationship extended beyond any potential family violence occurring, to the overall health of the patient and their family. Losing the relationship with the patient caused GPs and nurses great concern over the patient and their family’s wellbeing:

*'So sometimes we report things which wrecks our therapeutic relationship…But then, they* [child protection] *don’t do anything and it often feels like, well, why did I make that report? Because the family haven’t received any support and now, they’re not seeing me anymore.’* Zoe, GP, DiSS, Victoria.

Families became aware that they were being reported either because GPs or nurses decided to tell them in an effort to be transparent and maintain the therapeutic relationship, or the family deduced the GP or nurse reported as only they were aware of the family’s situation. Such was the fear and concern around losing the therapeutic relationship that a few GPs questioned the act of reporting:

*‘It's a really tricky question to say is that* [not reporting] *the wrong thing to do? Legally - the answer is definitely yes; in terms of child safety, probably still yes. In terms of what's best for the situation, because child safety is important of course, but there's more to it than that. Are you really going to blow that relationship?’* Charlie, GP, NSW.

In summary, GPs and nurses felt that even considering the possibility of child abuse was tantamount to betraying the trust that existed between them and their patient. This betrayal of trust was complex, occurring on multiple levels within the therapeutic relationship with the family and often resulting in the loss of that relationship.

### Riding the reaction wave

Managing patients’ reactions was a complex and difficult part of the response to child abuse. This was also a strong theme as most GPs and nurses in this study felt they had to ‘ride the wave’ (Daniel, GP, SA) of reaction in response to discussing child abuse. This theme details how participants managed their interaction with families suspected of experiencing child abuse, including the strategies they used, and the fear of retaliation they experienced on a personal, organisational and system level.

All participants wanted to maintain the therapeutic relationship, however, only some had tried and tested strategies. These participants used several strategies to manage the therapeutic relationship to maintain trust and avoid the conflict that resulted in a breakdown or loss of the relationship. Interestingly, all these strategies centred around GPs and nurses creating a shift in perception that they were part of a help-seeking process rather than a punitive system. This shift in perception was directed at both patients and GPs and nurses themselves. For example, some GPs and nurses framed reporting as an avenue of help-seeking to help them, as well as their patients, to feel more comfortable and less offended about reporting to child protection. Nora, a nurse responding to a young person who disclosed that they were thrown against a wall by their mother, used the ‘framing of reporting as an avenue for help-seeking’ strategy to convince the young person that reporting was necessary:

*‘…Kind of framing it* [reporting] *in a way that, ‘you told me for a reason, but we're going to make sure you get the help’.’* Nora, Nurse, DiSS, Victoria.

Others took advantage of the faceless and impersonal quality of the law and government to protect the relationship from conflict. In ‘blaming the government’, some participants created a common authority which placed them on the same side as the patient in relation to the government, thus encouraging the shift in perception. It should be noted that this strategy seemed to be used in contexts where patients had an existing distrust of the government, like Charlotte who reported working in an indigenous community feeling over-monitored by government agencies; or where patients stereotypically felt powerless in the face of authority, like Ben who worked with young people in DiSS:

*‘I have a thing - always blame the government because the government is this sort of nebulous entity, but they can't actually pin down and go and complain against.’* Charlotte, GP, NT.

*‘So, I called the medical defence and they said, yeah ‘you have to report it’ which I expected. So, I did it so it looked* [to the young person] *like it wasn't my decision, but it looked like my hands were tied.’* Ben, GP, DiSS, Victoria.

These strategies helped manage the conflict that arose and, in many cases, contributed to maintaining an ongoing positive therapeutic relationship. However, this was not always the case. Many feared retaliation from caregivers after discussing possible child abuse or reporting. Part of this fear was fuelled by the fact that GPs and nurses did not feel anonymous when reporting. One nurse reflected on how a report in a small community could ‘kill your standing’ (Daphne, Victoria) in that community. Because of this, GPs and nurses in this study often engaged in a conversation with caregivers about reporting. The conversation served two purposes: to provide transparency and maintain trust, and to warn caregivers so that they are not caught off guard if some action were to come from the report. However, having this conversation sometimes lead to a fear of retaliation on the GP or nurses’ part, even beyond the consulting room. Michaela, a nurse, described a family outing where she came into contact with a perpetrator she had reported to child protection:

*‘…then I’m at the swimming pool with my children and then the father is there with his children and I did feel a little bit vulnerable because he was aware that I had done it…Because my kids were in the same swimming class together. So, that was really confronting.’* Michaela, Nurse, DiSS, Victoria.

The fear of retaliation extended to colleagues and the clinic or organisation within which participants worked. Interestingly, this fear was expressed by nurses more than GPs. One nurse expressed concern about how a GP’s loyalty to a family might cause conflict between a nurse and a GP:

*‘…the other thing is the relationship that you would have with the rest of the practice if you report and they're not really happy that… What do you do if they* [the GP] *say, ‘no, you shouldn't, they're my friends…I know that family really well…’* Daphne, Nurse, Victoria.

Another nurse described a need for further support from her organisation around reporting child abuse:

*‘I want the organisation to support me in what I do…even though it's law, Government is protecting me, at the same time nobody wants to go into battle with the organisation.’* Shanika, Nurse, Victoria.

At the system level, some GPs and nurses feared retaliation from the law in their experience of responding to child abuse. The fear was based on the consequences of not reporting. Shanika speculated that jail time was a consequence, while one GP, Adam, spoke about the need to report to keep himself legally safe:

*‘I felt that I needed to, just to make sure that I was legally safe…I think there can be over-reporting because you’re afraid. You’re afraid that if you don’t report it and something does come out then you could get into trouble.’* Adam, GP, Victoria.

Overall, GPs and nurses had to ride a reaction wave that spanned from caregivers to colleagues to the law. GPs and nurses developed strategies to help them ride this reaction wave and maintain the therapeutic relationship. These strategies created perception shifts to make the act of reporting more tolerable for themselves and for their patients. Despite this, participants still feared retaliation from caregivers, as well as colleagues. Nurses were particularly concerned with how their standing in their organisation would be affected after a report. On a system level, some GPs and nurses used reporting as strategy to keep themselves legally safe.

### Opening a hornet’s nest

In addition to managing the therapeutic relationship, some GPs and nurses in this study also fell into the role of managing the relationship they and their patient had with the justice and child protection system (the system). By reporting to child protection, many GPs and nurses, particularly those operating within DiSS, felt they were going to ‘open a hornet’s nest’ (Bonnie, GP, Victoria) of complications which had to be managed. This theme reports how participants’ experiences with the child protection system highlighted the tension between professional ethical principles and system obligations. This is further explored by discussing the relationship between the system, patients and GPs and nurses.

Responding to child abuse by reporting was often viewed by participants as a violation of the common Hippocratic ethical principle ‘do no harm’. This principle forms a cornerstone of medical practice. However, many GPs and nurses witnessed harm being inflicted by the system on families and on their therapeutic relationship after making a report. Consequently, fulfilling mandatory reporting obligations made participants feel as if they were breaking their professional code of ethics. This caused participants acute discomfort, guilt and sometimes despair.

*‘…sometimes I’ve made a report knowing that it will have negative consequences just because you have to follow…Well, you’re supposed to ‘do no harm’, but you sometimes know that the intervention…is going to not be helpful.’* Jocelyn, GP, DiSS, Victoria.

*‘He* [the young person] *says that every time he tells somebody about what's happened, it comes back to bite him and then he gets in trouble for having said it…You have to weigh up whether telling somebody…is actually going to cause more harm in the end. That's a really big thing that I see over and over.’* Tara, GP, DiSS, Victoria.

Tara’s quote above illuminates an aspect of the relationship between young people and the system. Involving the system causes complications around the safety of the young person. In the eyes of DiSS GPs and nurses, young people are alert to the risks of reporting and are fearful of the effect of the system’s involvement on their relationship with the perpetrator. Despite mandatory reporting being aimed at securing child safety, some DiSS GPs and nurses saw that the potential to be reported discouraged some young people from disclosing and seeking help. Other young people sought help from GPs and nurses but tried to protect themselves from the risks by asking GPs and nurses not to report. In these circumstances, GPs and nurses felt caught between protecting young people and their reporting obligation.

*‘She* [the young person] *actually asked us not to call DHS* [child protection] *because it makes things worse…for her and she really didn’t want us to be involved in that…They* [young people] *were actively asking us not to do it* [report] *because they knew that they were going to be put at risk.’* Michaela, Nurse, DiSS, Victoria.

The view of some GPs and nurses that the system was incompetent further exacerbated the violation of the ‘do no harm’ principle. When the system failed to act in a protective and respectful manner towards young people, some GPs and nurses were compelled to account, and apologise to their patients, for system failures to minimise the harm caused. One GP, Isla, spoke of how a 16-year-old had disclosed sexual assault but was adamant that police not be involved. Isla was careful to emphasise this to child protection. However, Isla later became aware that the police had ambushed the young person at her workplace to confirm she did not want to press charges. Isla then described the effect this incident had on her relationship with the young person:

*‘She totally disengaged from me…I felt just so bad about that* [the police involvement] *and I did get her in, and I apologised, like I was really angry about it.’* Isla, GP, DiSS, Victoria.

As seen in the above quote, mediating the relationship between the system and patients occurred within a context of GPs’ and nurses’ personal feelings of frustration with the system. GPs and nurses in this study had to minimise or compartmentalise their feelings regarding the system when interacting with patients. Overall, most participants spoke about feeling ‘destroyed’ or ‘let down’ by the system. Others felt that their concerns were not being respected by the system:

*‘They* [child protection] *don't take it seriously. They don't understand why we might be concerned. They minimise the problem…That dismissive attitude is really patronising.’* Rose, GP, Victoria.

This quote demonstrates a parallel in the relationship GPs and nurses have with child protection and the relationship young people have with child protection. Some GPs and nurses in this study felt that their concerns, and the concerns of young people around their risk, were not taken seriously by the child protection system.

Overall, the response to child abuse required GPs and nurses to manage their relationship, and the relationship their patients had, with the system. At times, GPs and nurses felt the outcome of reporting, or lack thereof, caused further harm. Therefore, they sometimes felt they were compromising their code of ethics to fulfil their mandatory reporting obligation. This feeling was exacerbated when the system did not respond to their patients in a helpful way, and GPs and nurses were left to account and apologise for system failures beyond their control. In order to account and apologise, GPs and nurses had to compartmentalise their own feelings of frustration regarding the system.

### Battling emotions

The experience of the response to child abuse for all GPs and nurses in this study occurred upon an emotional battleground. The emotional battle was inseparable from the response itself and was linked to the underlying purpose of GPs’ and nurses’ profession:

*‘It's very hard to say ‘it's not for you to battle the emotional things, this is something that you've got to do’…If you don't battle that, you can't do this job…It's human related …. this is why we're in the profession. We do care about people, and we don't want to be wrong.’* Shanika, Nurse, Victoria.

This final theme explores how internal emotional battles led to some GPs and nurses experiencing burnout and vicarious trauma and led others to building resilience.

Burnout was an unsurprisingly common experience for most participants. Many participants felt a responsibility to ‘carry the story for people’ (Sophia, GP, DiSS, Victoria). The weight of these stories added to the emotional burden over time:

*‘Although, I'm finding stuff is building up more and I don't know if it's over the years…I'm finding myself more affected than I used to be by some of the stories.’* Bonnie, GP, Victoria.

As GP and nurse participants battled the growing emotional burden, they found themselves ‘compartmentalising’ (Tara, GP, DiSS, Victoria) the battle to enable them to respond to new or exacerbated child abuse. However, compartmentalising appeared to only increase the amount of burnout being felt, rather than assisting the situation. A few participants became so burnt out that they decided to focus on other areas of their practice that were comparatively less emotionally challenging:

*‘I am actually working in some different areas now. I’m working in a weight management clinic to have a bit of space from that type of work and to get the balance back for me.’* Sophia, GP, DiSS, Victoria.

Some participants experienced a greater emotional response to some stories than others. In these situations, burnout was experienced hand-in-hand with vicarious trauma. For some participants, this vicarious trauma occurred as an emotional response to the trauma inflicted on their patient. For example, Tara, at the time of the interview, had recently had a young patient take their own life. Tara was in a fragile emotional state as she described the enormity of her helplessness and the associated emotional risks that seeped into her practice:

‘…*it's also risky emotionally, because you go, ‘okay, every single kid that I treat, what is their risk? How many dead bodies am I going to have floating around me?”* Tara, GP, DiSS, Victoria.

For other participants, vicarious trauma arose when their own experience of child abuse was triggered. Adam was an example of this. He described the child abuse experience of one of his older patients. However, part way, he became emotional and couldn’t speak. Later in the interview, he reflected on his reaction:

*‘Occasionally you get a little emotional with patients…I don’t know why this one - it’s not as if I was - I had a couple of strappings by dad but I would never have said I was abused by my parents but for some reason it just hits me, that one.’* Adam, GP, Victoria.

Adam, while drawing parallels between his and his patient’s experience, does not label his experience as abuse and yet cannot account for why he struggles emotionally with this patient’s story.

The psychological effects of experiencing burnout and vicarious trauma were immense for many GPs and nurses. Some participants described feeling physically ill or feeling as if their ‘guts are churning’ (Lilly, Nurse, DiSS, Victoria) after listening to abuse experiences or making the decision to report. Others spoke of experiencing depression or feeling as if they were ‘covered in toxic waste’ (Tara, GP, DiSS, Victoria) after responding to child abuse. Some of these effects are exemplified in a group interview with three GPs working within DiSS. Jocelyn described a situation of major abuse in limited detail. She finished her account by stating her reasons for not providing more specific details. Isla and Heidi then expressed their reaction to listening to more detail:

[Isla*: it just makes me sick*] *…Oh, it’s even worse than what I said because I didn’t want to say - you don’t want to know the other stuff.* [Heidi*: Oh, I don’t even want to know, don’t even tell me. I’m so disturbed by that.*] [Isla: *Bad dreams, bad dreams*]*.’* Jocelyn, GP, DiSS, Victoria; Isla, GP, DiSS, Victoria; Heidi, GP, DiSS, Victoria.

Isla’s comment ‘bad dreams’ indicates that she has experienced nightmares relating to the child abuse to which she has had to respond. It is a testament to how emotionally challenging this work can be.

Despite these emotional challenges, some participants found positive aspects of their response to child abuse that helped build resilience, including personal strengths:

*‘I would read the children's statements before I saw them. They were often quite sickening…. But what I could really do was really put the child and the family member at ease... I think in a sense if I feel like I'm doing good I can tackle almost anything.’* Penelope, GP, NSW.

Others drew resilience from their patient’s example:

*‘I think observing what has made them survivors is what’s helped to give me strength.’* Lalita, GP, SA.

It is important to note that those participants who displayed a sense of resilience seemed to convey less emotional distress during the interview. They appeared to diminish their experiences of emotional conflict by focusing on the good.

Overall, GPs’ and nurses’ experience of the response to child abuse was characterised by an emotional battle. Burnout was commonly experienced and was sometimes punctuated with vicarious trauma. Vicarious trauma was experienced either in response to hearing traumatic experiences or because of triggering of personal previous experience with child abuse. However, some participants experienced reliance-building aspects within the response to child abuse.

## Discussion

In this study, GPs’ and nurses’ experience of the response to child abuse was characterised by four themes: *blowing trust out of the water; riding the reaction wave; opening a hornet’s nest;* and *battling emotions.* The inductive findings of this study resonated with aspects of emotional labour theory. Thus, this discussion will position the themes in relation to the theory of emotional labour. Emotional labour played a large part in the response to child abuse and occurred across three levels for GPs and nurses in this study: internal, organisational, and systemic.

GPs and nurses engaged in internal emotional labour where they managed their internal emotions. In *‘blowing trust out of the water’*, they had to suppress their fear of betraying trust in order to broach the topic of child abuse. They also had to manage their feelings around conflicting priorities in the therapeutic relationship. In *riding the reaction wave,* GPs and nurses had to engage in surface acting when they feared retaliation from caregivers and colleagues. In *opening a hornet’s nest and battling emotions*, GPs and nurses had to push through burnout to minimize their frustration with the system when interacting with patients. In more intense cases of burnout and frustration, GPs and nurses engaged in surface-acting as a means to push through burnout and continue performing. Brotheridge, who compared emotional labour and burnout in several professions, found that engaging in surface acting increased burnout [20]. This finding was echoed in other studies by Montgomery et al. and Soni [23, 32]. Hence, some GPs and nurses may find themselves caught in a cycle of increasing burnout by using surface acting to continue performing through burnout. Given our findings, we propose that the cycle of increasing burnout can be partly countered by focusing on positive aspects of the response to child abuse. We speculate that this may be because these resilience-building aspects are the response elements that align with the professional goal of providing whole person care [33].

Organisational emotional labour was emotion work that was used to manage the therapeutic relationship, as seen in *riding the reaction wave*. Through this emotion work, GPs and nurses developed strategies that were centred around creating shifts in perception, including framing reporting as an avenue for help-seeking and blaming the government. In convincing themselves and their patients that reporting was an avenue of help-seeking, some GPs and nurses were engaging in deep acting. Both Brotheridge and Soni found that engaging in deep acting was not associated with emotional exhaustion and contributed to a greater sense of personal accomplishment in participants’ profession [20, 23]. In the present study, GPs and nurses used deep acting to align their professional goal of providing care with their mandatory reporting obligations. Although this may have made the task of mandatory reporting emotionally easier at the time of the report, some participants still witnessed harm following their report which highlighted the existing tension between professional ethics and reporting. Thus, deep acting may not have been as effective here as was described in other studies [20, 23].

In fact, the misalignment of goals between the professional ethics and mandatory reporting legislation, as described in *opening a hornet’s nest*, is the aspect of the study that resonates most with emotional labour theory. In addition to subscribing to the principle ‘do no harm’, general practice aims to provide care for the entire family by prioritising and valuing the therapeutic relationship [34]. From GPs’ and nurses’ perspective, the child protection system has the goal of protecting children through mandatory reporting of child abuse [11]. However, because reporting can disrupt the therapeutic relationship and may increase harm towards a family, participants often found that their internal and professional goals were in direct conflict with the goals of the system. This conflict leads to emotive dissonance which occurs when there is a conflict between internal and organisational goals or feeling states [19, 21].

Noting that this definition of emotive dissonance describes it as occurring across two levels – internal and organisational – the current study reveals participants experienced emotive dissonance across three levels: internal, organisational (where the organisation is general practice or DiSS) and systemic (where the system is the child protection system). Thus, the current study identifies the novel concept of systemic emotional labour. To allay the emotional turmoil experienced in the response to reporting, participants engaged in deep acting, such as re-framing their thought process around reporting, or surface acting, when they apologised for the failings of the system. However, because of the continued misalignment between internal feeling states, organisational goals and systemic goals, GPs and nurses may be in a constant state of emotive dissonance when responding to child abuse, which is then heighted when the system does not respond appropriately. This emotive dissonance then has the potential to translate into system-induced burnout.

There were several limitations to this study. First, this study explored the response to child abuse within DiSS, which is in an uncommon practice setting for GPs and nurses in Australia. While the themes presented in this paper were across all participants, some themes (e.g. *opening a hornet’s nest)* were more pertinent to DiSS participants. Further, the majority of the participants in this study were GPs who practiced within metropolitan settings in Victoria and few were of a culturally diverse background. Therefore, these themes may not resonate with a broader geographical or cultural population of GPs and nurses. Additionally, recruitment for this study may have attracted GPs and nurses who had an existing interest in family violence, which may introduce self-selecting bias.

However, to the authors’ knowledge, this is the first study to undertake an in-depth and qualitative exploration into how GPs and nurses experience the response to child abuse in Australia. It is also the first study to explore the response to child abuse in-depth within the context of emotional labour, particularly the use of emotional labour to manage therapeutic relationships in primary care settings.

The findings from this study have several implications for GPs and nurses in clinical practice. First, this study highlights the importance of GPs and nurses being aware of how they are managing their internal feeling states while practicing, as this awareness can lead to opportunities to reduce burnout. Second, the strategies identified in *riding the reaction wave* to manage the therapeutic relationship during the reporting process can be adapted by GPs and nurses when responding to child abuse. However, further research is needed to explore other strategies GPs and nurses may use and the efficacy of these strategies. Third, this study highlights issues relating to GP and nurse safety within the response to child abuse. It is important for GPs and nurses to be aware of potentially unsafe or confronting situations and safety plan accordingly, especially for those who practice in small close communities.

Organisations can encourage this safety planning by promoting safety policies and fostering an environment that prioritises the safety and emotional wellbeing of staff. For example, reducing time and financial pressure on GPs and nurses when dealing with a possible abuse case, encouraging interpersonal relationships within the practice or modifying the workplace environment according to a model of general practice wellbeing [35]. Additionally, these actions can be extended to enable GPs and nurses to feel secure in their decision to report, even if there is disagreement about reporting within the practice. It is important for organisations, as well as GPs and nurses, to be aware of the tension between professional ethical principles and mandatory reporting obligations and to reflect on how it may affect their response to child abuse. An internal, organisational and systemic values assessment may aid these reflections. We recognise that the implications listed above for individuals and organisations may seem paltry and will not solve the complex issues surrounding the response to child abuse identified in this paper. Rather, we suggest that these implications may form part of a suite of strategies and resources professionals may use to manage the complexities and emotional demands of the response to child abuse within a system that is clearly in need of major reform. Thus, these considerations may also benefit policy makers and the child protection system, especially regarding the alignment of reporting obligations with the goal of protecting children. Given the complexity of responding to child abuse, some authors have questioned the validity of mandatory reporting and condemned current structuring of child protection systems in Western countries [36]. Our findings contribute to this debate. A potentially well-functioning aspect of Western child protection systems is mandated reporters ability to ‘test’ their case anonymously with child protection to determine if it meets the reportable threshold [37]. While this service is theoretically available in Australia, it is not consistently offered across all regions. If the Australian child protection system could be additionally resourced to uniformly provide this service to GPs and nurses, it may reduce systemic emotional labour that is currently undertaken by GPs and nurses and may therefore reduce system-induced burnout.

## Conclusion

GPs’ and nurses’ experience of responding to child abuse in this study is characterised by a complex interplay of emotional state management and relationship management. Participants felt that they were betraying the trust of the therapeutic relationship and thus, had to manage their patients’ reactions to preserve the therapeutic relationship when navigating the response to child abuse. Additionally, they took on the role of managing the relationship they and their patients had with the child protection system. Participants also felt they had to compromise their professional ethical principles to fulfil their mandatory reporting obligations. Thus, participants experienced an internal emotional battleground, leading to some experiencing burnout or vicarious trauma and others resilience. Participants undertook emotional labour across three levels during their response to child abuse: internal, organisational and systemic. Being aware of emotional labour, conducting values assessments and developing strategies to manage emotional states and relationships in a healthy way may be beneficial to GPs and nurses responding to child abuse. These findings could inform organisations that support and train primary care professionals.

## Data Availability

The authors declare that data supporting the findings of this study are available within the article.

## References

[1] World Health Organization (2020). Global status report on preventing violence against children.

[2] Australian Institute of Health and Welfare (2020). Australia's children.

[3] Gilbert R, Kemp A, Thoburn J, Sidebotham P, Radford L, Glaser D (2009). Recognising and responding to child maltreatment. Lancet.

[4] Hart H, Rubia K (2012). Neuroimaging of child abuse: a critical review. Front Hum Neurosci.

[5] Vink RM, van Dommelen P, van der Pal SM, Eekhout I, Pannebakker FD, Velderman MK (2019). Self-reported adverse childhood experiences and quality of life among children in the two last grades of Dutch elementary education. Child Abuse Negl.

[6] Felitti VJ (1998). Relationship of childhood abuse and household dysfunction to many of the leading causes of death in adults : the adverse childhood experiences (ACE) Study. Am J Prev Med.

[7] Pournaghash-Tehrani SS, Zamanian H, Amini-Tehrani M. The impact of relational adverse childhood experiences on suicide outcomes during early and young adulthood. J Interpers Violence. 2019. 10.1177/0886260519852160. 10.1177/0886260519852160 31142213

[8] Salokangas RK, Schultze-Lutter F, Schmidt SJ, Pesonen H, Luutonen S, Patterson P, et al. Childhood physical abuse and emotional neglect are specifically associated with adult mental disorders. J Ment Health. 2020;29(4):376–84. Available from: https://discovery.ebsco.com/linkprocessor/plink?id=c44be90e-839c-3cb5-aaa1-88a408b230af. [cited 2022 Mar 15]. 10.1080/09638237.2018.1521940 30675805

[9] Rivara FP, Anderson ML, Fishman P, Bonomi AE, Reid RJ, Carrell D (2007). Intimate partner violence and health care costs and utilization for children living in the home. Pediatrics..

[10] Australian Institute of Health and Welfare. Australia's health: AIHW; 2020 [Available from: https://www.aihw.gov.au/reports/australias-health/primary-health-care.

[11] Wilson IA, Lee J (2021). Barriers and facilitators associated with child abuse and neglect reporting among child care professionals: a systematic review. J Psychosoc Nurs Ment Health Serv.

[12] Mathews B. Mandatory reporting laws for child sexual abuse in Australia: a legislative history. 2014. Sydney: Royal Commission into Institutional Responses to Child Sexual Abuse. Available at http://www.childabuseroyalcommission.gov.au/documents/royal-commission-report-ben-mathews-for-rc-publica.

[13] McTavish JR, Kimber M, Devries K, Colombini M, MacGregor JC, Wathen CN (2017). Mandated reporters’ experiences with reporting child maltreatment: a meta-synthesis of qualitative studies. BMJ Open.

[14] Francis K, Chapman Y, Sellick K, James A, Miles M, Jones J (2012). The decision-making processes adopted by rurally located mandated professionals when child abuse or neglect is suspected. Contemp Nurse.

[15] Kuruppu J, McKibbin G, Humphreys C, Hegarty K (2020). Tipping the scales: factors influencing the decision to report child maltreatment in primary care. Trauma Violence Abuse.

[16] Demircin S, Tutunculer A, Aslan F, Velipasaoglu Guney S, Atilgan M, Gulkesen H (2017). The knowledge level and opinions of physicians about the medical and legal procedures related to physical child abuse. Balkan Med.

[17] Flaherty EG, Jones R, Sege R (2004). Telling their stories: primary care practitioners' experience evaluating and reporting injuries caused by child abuse. Child Abuse Negl.

[18] Kuruppu J, Forsdike K, Hegarty K (2018). 'It's a necessary evil': experiences and perceptions of mandatory reporting of child abuse in Victorian general practice. Austr J Gen Pract.

[19] Hochschild AR (2012). The managed heart. [electronic resource] : commercialization of human feeling.

[20] Brotheridge CM, Grandey AA (2002). Emotional labor and burnout: comparing two perspectives of “people work”. J Vocat Behav.

[21] Jackson S, Backett-Milburn K, Newall E (2013). Researching Distressing Topics:Emotional Reflexivity and Emotional Labor in the Secondary Analysis of Children and Young People’s Narratives of Abuse. SAGE Open.

[22] Tarzia L (2021). Women’s emotion work in the context of intimate partner sexual violence. J Fam Violence.

[23] Soni S (2017). Workplace emotions: a study of frontline hotel employees. Manage Labour Stud.

[24] Kulkarni S, Bell H, Hartman JL, Herman-Smith RL (2013). Exploring individual and organizational factors contributing to compassion satisfaction, secondary traumatic stress, and burnout in domestic violence service providers. J Soc Soc Work Res.

[25] Trippany RL, White Kress VE, Wilcoxon SA (2004). Preventing vicarious trauma: what counselors should know when working with trauma survivors. J Counsel Dev.

[26] Creswell JW, Creswell JW (2013). Qualitative inquiry & research design : choosing among five approaches.

[27] Laverty SM (2003). Hermeneutic phenomenology and phenomenology: a comparison of historical and methodological considerations. Int J Qual Methods.

[28] Van Manen M. Researching lived experience : human science for an action sensitive pedagogy: State University of New York Press; 1990.

[29] Corbin J, Morse JM (2003). The unstructured interactive interview: issues of reciprocity and risks when dealing with sensitive topics. Qual Inq.

[30] Flick U. The Sage handbook of qualitative data collection: Sage; 2017.

[31] Braun V, Clarke V. Reflecting on reflexive thematic analysis. Qual Res Sport Exerc Health. 2019;11(4):589–97.

[32] Montgomery AJ, Panagopolou E, de Wildt M, Meenks E. Work-family interference, emotional labor and burnout. J Manag Psychol. 2006.

[33] Royal Australian College of General Practitioners (2019). What is General Practice?.

[34] Royal Australian College of General Practitioners (2016). The five domains of general practice: a comprehensive, robust framework.

[35] Prentice S, Benson J, Dorstyn D, Elliott T (2022). Wellbeing conceptualizations in family medicine trainees: a hermeneutic review. Teach Learn Med.

[36] Melton GB (2005). Mandated reporting: a policy without reason. Child Abuse Negl.

[37] Family and Community Services (2019). Mandatory reporters: what to report and when 2019.

